# Indole-3-Carbinol Selectively Prevents Chronic Stress-Induced Depression-but not Anxiety-Like Behaviors via Suppressing Pro-Inflammatory Cytokine Production and Oxido-Nitrosative Stress in the Brain

**DOI:** 10.3389/fphar.2022.829966

**Published:** 2022-02-15

**Authors:** Shengying Pan, Yaoying Ma, Rongrong Yang, Xu Lu, Qingsheng You, Ting Ye, Chao Huang

**Affiliations:** ^1^ Department of Neurology, The People’s Hospital of Danyang, Affiliated Danyang Hospital of Nantong University, Danyang, China; ^2^ Department of Pharmacology, School of Pharmacy, Nantong University, Nantong, China; ^3^ Department of Anesthesiology, Affiliated Hospital of Nantong University, Nantong, China; ^4^ Department of Cardiothoracic Surgery, Affiliated Hospital of Nantong University, Nantong, China

**Keywords:** indole-3-carbinol, depression, anxiety, neuroinflammation, oxido-nitrosative stress

## Abstract

Indole-3-carbinol (I3C), a phytochemical enriched in most cruciferous vegetables, has been shown to display various biological activities such as anti-oxidative stress, anti-inflammation, and anti-carcinogenesis. In this study, we investigated the regulatory effect of I3C on chronic stress-induced behavioral abnormalities in mice. Results showed that repeated I3C treatment at the dose of 10, 30, and 60 mg/kg prevented chronic social defeat stress (CSDS)-induced behavioral abnormalities in the tail suspension test, forced swimming test, sucrose preference test, and social interaction test in mice, and did not affect CSDS-induced behavioral abnormalities in the elevated plus maze, light-dark test, and open-field test, suggesting that the I3C treatment selectively prevents the onset of depression- but not anxiety-like behaviors in chronically stressed mice. Further analysis demonstrated that repeated I3C treatment (60 mg/kg, 10 days) prevented CSDS-induced increases in levels of interleukin-1β (IL-1β), IL-6, and tumor necrosis factor-α (TNF-α) mRNA and protein, but did not affect CSDS-induced decreases in levels of IL-4, IL-10, and Ym-1 mRNA and/or protein in the hippocampus and prefrontal cortex, suggesting that I3C can selectively prevent chronic stress-induced pro-inflammatory but not anti-inflammatory responses in the brain. Further analysis showed that repeated I3C treatment (60 mg/kg, 10 days) prevented CSDS-induced increases in levels of nitrite and malondialdehyde (MDA), decreases in contents of glutathione (GSH), and decreases in levels of brain derived neurotrophic factor (BDNF) protein in the hippocampus and prefrontal cortex. These results demonstrated that I3C selectively prevents chronic stress-induced depression-like behaviors in mice likely through suppressing neuroinflammation and oxido-nitrosative stress in the brain.

## Introduction

Social stress exposure is a common phenomenon in the modern society, which can lead to the development of psychological disorders, such as depression and anxiety (Toyoda, 2017; Wang et al., 2021). Clinically-available drugs for the management of psychological disorders are known to display numerous detrimental effects, such as sleep disturbance and an increased risk for suicide (Robillard et al., 2016; Niculescu et al., 2017), and only a small part of patients receiving anti-psychiatric drugs acquire excellent therapeutic effects (Fitzgerald and Bronstein, 2013; Sonmez et al., 2020). Thus, it is necessary to develop novel drugs for the treatment of psychological disorders.

Increased neuroinflammatory response is a novel hypothesis for the explanation of the pathogenesis of psychologcial disorders (Dantzer, 2006; Kubera et al., 2011; Ramirez et al., 2017; Weber et al., 2017; Dantzer, 2018; Chaves-Filho et al., 2019; Bekhbat et al., 2022). Researchers have observed high levels of pro-inflammatory cytokines including interleukin-1β (IL-1β), IL-6, and tumor necrosis factor-α (TNF-α) in the blood of patients suffering from depression and anxiety (Tang et al., 2018; Petralia et al., 2020; Primo de Carvalho Alves and Sica da Rocha, 2020). In rodent models of depression and anxiety, high levels of pro-inflammatory cytokines and low levels of anti-inflammatory mediators such as IL-10 and IL-4 in the brain are also correlated with the progression of abnormal behaviors (Wang et al., 2018; Walker et al., 2019; Zhang et al., 2019; Guha et al., 2020; Wu et al., 2021). Skewing the neuroinflammatory responses in the brain towards an anti-inflammatory state can prevent the progression of abnormal behaviors in animals stimulated with detrimental stress (Zhao et al., 2016; Duan et al., 2020; Jing Li et al., 2020; Wu et al., 2020; Kumar et al., 2021). Moreover, direct infusion of pro-inflammatory cytokines such as IL-1β and interferon-α (INF-α) into the brain in animals can also induce behavioral abnormalities (Park et al., 2015; Zhifei Li et al., 2020), and clinically-available antidepressants such as fluoxetine can improve behaviors in depressed animals (Du et al., 2016) and patients suffering from generalized anxiety disorders (Hou et al., 2019) partially through suppression of inflammation. Thus, searching drugs that can suppress neuroinflammation could be a potential strategy for the prevention of psychological disorders.

Indole-3-carbinol (I3C) is a phytochemical present at a relatively high level in most cruciferous vegetables, such as cabbage, broccoli, and collard greens. Functional studies have shown that I3C supplementation can produce numerous pharmacological activities, such as anti-oxidative stress and anti-carcinogenesis (Verhoeven et al., 1997; Hajra et al., 2018; Saini et al., 2020). I3C supplementation can also suppress the production of pro-inflammatory cytokines in different animal models of disease. For example, its supplementation has been shown to ameliorate 1) carbon tetrachloride-induced acute liver injury (Munakarmi et al., 2020), 2) colitis-induced colonic pro-inflammatory responses and microbial dysbiosis (Busbee et al., 2020), and Complete Freund’s Adjuvant-induced arthritis (Hasan et al., 2018) via similar down-regulatory effects on inflammation. In a recent study, I3C supplementation was found to protect the retina against the light-damage-induced degeneration partially through inhibiting the over-activation of microglia (Khan and Langmann, 2020). Here, we speculate that I3C supplementation may affect the progression of depression- and anxiety-like behaviors in chronically stressed mice by altering the brain’s responses to neuroinflammation and oxidative stress. This hypothesis was examined by s series of behavioral tests, real-time reverse transcriptase-PCR, and biochemical measurements.

## Materials and Methods

### Materials

I3C, which was purchased from Sigma (Saint Louis, MO, United States), was firstly dissolved in dimethyl sulfoxide (DMSO) at a concentration of 100 mg/ml, and then the olive oil was added into the I3C-containing solutions in a 10:1 ratio. The stock solution was stocked at −20°C. The other agents were purchased from commercial suppliers.

### Animals

The six-week-old male C57BL6/J mice (body weight: 22–26 g) and eight-week-old male and female CD1 mice (each number is 100, body weight: 30–35 g) were purchased from Beijing Vital River Laboratory Animal Technology Co., Ltd. (Beijing, China). The female CD1 mice were used to induce aggressive behaviors in male CD1 mice. The male CD1 resident mice were found to attack the intruder mice when a male C57BL6/J intruder came in and a female CD1 sexual partner was removed (Goyens and Noirot, 1975). Before the construction of depression model, one male and one female CD1 mouse were housed together, and the C57BL6/J mice were housed 5 per cage. All mice were kept at conditions with 12-h light/dark cycle (lights on from 07:00 to 19:00), 23 ± 1°C ambient temperature, 55 ± 10% relative humidity, and free access to food and water. The total numbers of mice for behavioral and biochemical evaluations and analysis are 304. Animal experiments were approved by the University Animal Ethics Committee of Nantong University (Permit Number: 2110836) and conducted according to internationally accepted guidelines for the use of animals in toxicology as adopted by the Society of Toxicology in 1999.

### Procedures for Drug Treatment, Behavioral Tests, and Biochemical Measurement

**FIGURE 1:**
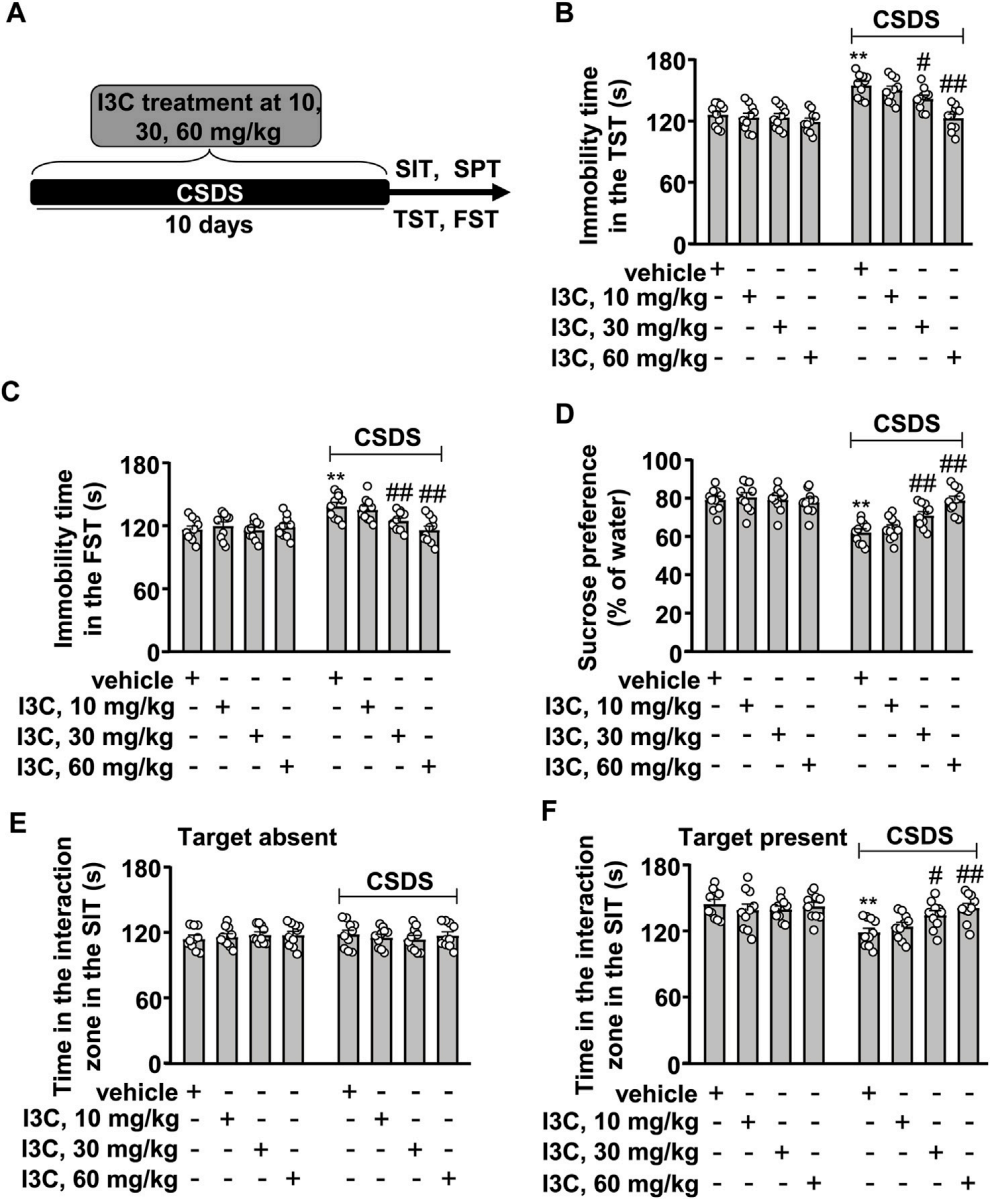
Dose-dependent effect of I3C on CSDS-induced depression-like behaviors in mice. **(A)** A schematic diagram showing the timeline for the evaluation of the effect of I3C treatment at different doses on CSDS-induced depression-like behaviors in mice. **(B,C)** Quantitative analysis showing the effect of I3C treatment (10, 30, and 60 mg/kg) on CSDS-induced increases in the immobility time in the TST **(B)** and FST **(C)** in mice (*n* = 10, **p < 0.01 vs. vehicle; #*p* < 0.05 or ##*p* < 0.01 vs. vehicle + CSDS). **(D)** Quantitative analysis showing the effect of I3C treatment (10, 30, and 60 mg/kg) on CSDS-induced reductions in sucrose intake in the SPT (*n* = 10, **p < 0.01 vs. vehicle; ##*p* < 0.01 vs. vehicle + CSDS). **(E,F)** Quantitative analysis showing the effect of I3C treatment (10, 30, and 60 mg/kg) on CSDS-induced changes in the time spent in the interaction zone in the SIT (target absence: **(E)**; target presence **(F)**; *n* = 10, **p < 0.01 vs. vehicle; #*p* < 0.05 or ##*p* < 0.01 vs. vehicle + CSDS). Data are shown as mean ± SEM.

The I3C was prepared immediately before use and delivered intraperitoneally once daily in a volume of 150 μl per mouse at 8 a.m. In experiments for the evaluation of depression-like behaviors in animals, a separate cohort of stress-naïve and chronically stressed mice were assigned into eight groups with vehicle or 10, 30, or 60 mg/kg of I3C treatment (10 in each group, total number: 80). The social interaction test (SIT), tail suspension test (TST), and forced swimming test (FST) were conducted on the first, second, and third day, respectively, after the discontinuation of CSDS stimulation, during which the I3C was still given, aiming to keep sustained actions of I3C. To avoid its potential influence to animal phenotype, the sucrose preference test (SPT) was conducted in a separate group of mice after the discontinuation of CSDS stimulation, during which the I3C was also given continually once daily. The schematic diagrams for the concrete arrangement for depression-like behavior assays and drug treatment were shown in Figure 1A.

**FIGURE 2:**
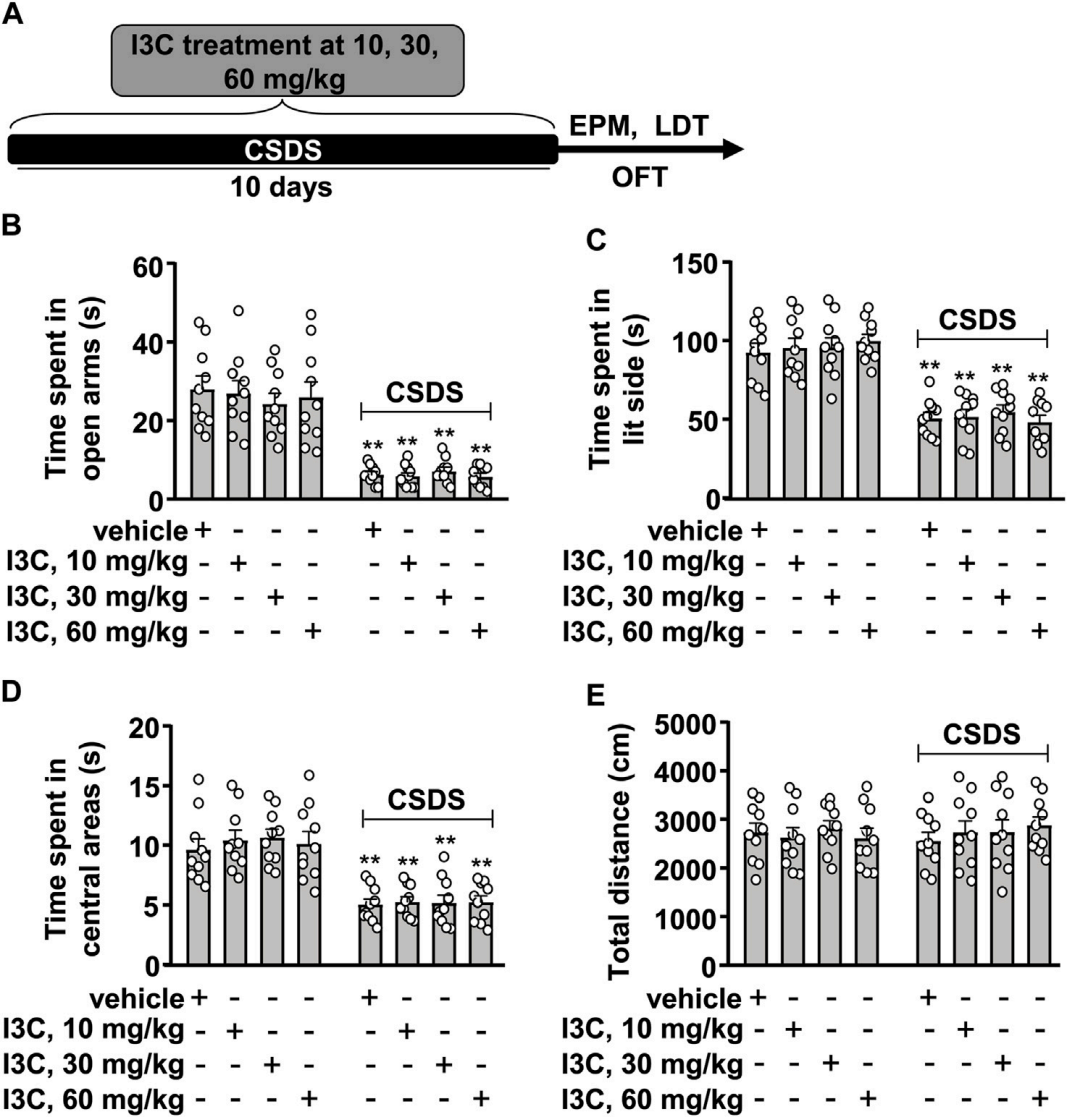
Dose-dependent effect of I3C on CSDS-induced anxiety-like behaviors in mice. **(A)** A schematic diagram showing the timeline for the evaluation of the effect of I3C treatment at different doses on CSDS-induced anxiety-like behaviors in mice. **(B‒D)** Quantitative analysis showing the effect of I3C treatment (10, 30, and 60 mg/kg) on CSDS-induced decreases in the time spent in open arms in the EPM [**(B)**, *n* = 10], in lit side in the LDT [**(C)**, *n* = 10], and in central areas in the OFT [**(D)**, *n* = 10)] in mice (**p < 0.01 vs. vehicle). **(E)** Quantitative analysis showing the effect of I3C treatment (10, 30, and 60 mg/kg) on the total distance of mice treated with or without CSDS in the OFT (*n* = 10). Data are shown as mean ± SEM.

Similarly, in experiments for the evaluation of anxiety-like behaviors, a separate cohort of stress-naïve and chronically stressed mice were assigned into eight groups with vehicle or 10, 30, or 60 mg/kg of I3C treatment (10 in each group, total number: 80). The elevated pluz maze (EPM) test, light-dark test (LDT), and open field test (OFT) were conducted on the first, second, and third day, respectively, after the discontinuation of CSDS stimulation, during which the I3C was given once daily, aiming to keep sustained actions of I3C. The schematic diagrams for the concrete arrangement for anxiety-like behavior assays and drug treatment were shown in Figure 2A.

Researchers were blinded to the group allocation and data analysis. Behavioral tests were conducted during the light phase and each group consisted of 10 mice. To avoid the possible influence of the behavioral assays to molecular analysis, we used the other animals, which were allocated into 4 groups (vehicle, I3C, CSDS, and I3C + CSDS) and consisted of 8 mice in each group, to prepare the brain tissues for the further real-time PCR and biochemical measurements. Throughout the experiment, the mice were transported in their home cage, and during the test, they were kept transiently in a new cage in the testing room. The environment in the testing room was the same as that in the housing room.

### Chronic Social Defeat Stress

The CSDS model was constructed according to previous studies (You et al., 2017; Song et al., 2018). To keep attack intensity as consistent as possible, one week before CSDS exposure, the eligibility of aggressive CD1 mouse (male, *n* = 100) was selected by following criteria for 3 days: the latency of CD1’s first attack was *<* 90 s but longer than 5 s, and the CD1 mouse attacked for at least 2 consecutive days during the 3 days selected process. During CSDS exposure, each C57BL6/J mouse, which is considered the subjects, was exposed to a novel aggressive CD1 mouse each day for up to 10 min, and then C57BL6/J mice were separated from the CD1 aggressor by plastic dividers with holes during the next 24 h. This procedure persisted 10 days. In order to avoid physical wounds, plastic dividers were set when C57BL6/J mice displayed submissive behavior, which include immobility, trembling, crouching, fleeing, and an upright posture. Control mice were housed in similar partitioned cages with another mouse from the same genotype and were handled daily throughout 10-day protocol period.

### SIT

The SIT, conducted according to previous studies (You et al., 2017; Song et al., 2018), comprises a first 5 min trial in the absence of the aggressor, immediately followed by an additional 5 min trial in the presence of the caged aggressor. In the first trial with target absent, each mouse was placed into an open-field apparatus and allowed to explore a plastic enclosure placed with the pre-defined interaction zone (a novel area with a small animal cage at the one end of the open-field apparatus). In the second trial with target present, each mouse was returned to the open-field arena containing a plastic enclosure now holding an unfamiliar CD1 mouse. The amount of time in the interaction zone was obtained using Ethovision XT (Noldus, United States) software. The apparatus in the SIT was cleaned thoroughly with 70% ethanol after each trial to remove olfactory cues.

### TST

The TST was conducted according to previous studies (Gu et al., 2021). The mice were habituated to the testing room for 20 min before the session started and then suspended 50 cm above the floor for 6 min by adhesive tape placed approximately 1 cm from the tip of the tail. The mice were considered immobile when they hung passively and were completely motionless (any mouse that climbed its tail was excluded from further analysis). The duration of immobility during the last 4 min of suspension was recorded by a video (Anhui Zhenghua Biological Instrument Equipment Co. Ltd., Huaibei, China).

### FST

The FST was conducted according to previous studies (Porsolt et al., 1977; Gu et al., 2021). The mice were habituated to the testing room for 20 min before the session started and then placed in a clear glass cylinder (height in 25 cm and diameter in 10 cm) filled to 10 cm with water at 25 ± 1°C for 6 min and defined immobile when they floated in the water without struggling, making only necessary movement to keep their heads above the water. The duration of immobility during the last 4 min of forced swimming was recorded by a video (Anhui Zhenghua Biological Instrument Equipment Co. Ltd., Huaibei, China).

### SPT

The SPT was conducted according to our previous studies (Liu et al., 2017a; Liu et al., 2017b). Mice were given the choice to drink from two bottles in individual cages, one with 1% sucrose solution and the other with water, and were acclimatized to the two-bottle choice condition for 2 days. The position of the two bottles was changed every 6 h to prevent side preference. On the testing day after a 24 h-deprivation of food and water, mice were firstly habituated to the testing room for 20 min before the session started, and then exposed to pre-weighed bottles for 6 h with their position interchanged. The sucrose preference was calculated as a percentage of the consumed sucrose solution relative to the total amount of liquid intake.

### EPM

The EPM test was performed according to one of our previous studies (Li et al., 2020). The apparatus under illumination (80 lx) comprised of two opposite-facing closed arms (300 (D) × 50 (W) × 150 (H) mm), two opposite-facing open arms (300 (W) × 50 (D) mm), and a central area (50 (D) × 50 (W) mm), which were raised 50 cm above ground by a base. Before test, mice were habituated to the testing room for 20 min. During test each mouse was put in the center region facing towards the open arm. The time spent by each mouse in open arms was recorded for 5 min with a video camera (Anhui Zhenghua Biological instrument equipment Co. Ltd., Huaibei, China) and scored as exploratory behaviors. The apparatus in the open and closed arms was cleaned thoroughly with 70% ethanol after each trial to remove olfactory cues.

### LTD

The LTD was performed according to previous studies using an apparatus consisting of two glass boxes (27 × 21 × 24 cm) with an interconnecting grey plastic tunnel (7 × 10 cm) (Zhu et al., 2018; Zhu et al., 2020). One of the boxes was lit by a 60-W desk lamp (400 lx) placed 30 cm above the box, providing the only laboratory illumination, and the other box was painted black and was weakly lit by a red 25-W bulb (0 lx). The floor was lined into 9 cm squares. Before test the mice were habituated to the testing room for 20 min. During test each mouse was introduced into the black side and their times spent in lit side were recorded during the 5 min-observation with a video camera (Anhui Zhenghua Biological instrument equipment Co. Ltd., Huaibei, China) and scored as exploratory behaviors. The apparatus in the lit and dark box was cleaned thoroughly with 70% ethanol after each trial to remove olfactory cues.

### OFT

The OFT was performed according to one of our previous studies (Li et al., 2020). Mice were habituated to the testing room for 20 min before the session started in a dimly environment illuminated with a red bulb (50 W) on the ceiling. During the test, each mouse, which was initially placed at the center region, was allowed to travel freely in a cubic chamber (360 (W) × 360 (H)×360 (D) mm) for 15 min. The time spent in the central area and the total distance of mice in the open field were recorded with the automated analyzing system (Anhui Zhenghua Biological Instrument Equipment Co. Ltd., Huaibei, China). The apparatus in the open field was cleaned thoroughly with 70% ethanol after each trial to remove olfactory cues.

### Real-Time Reverse Transcriptase-PCR

Immediately after the discontinuation of behavioral assays, mice were sacrificed by rapid decapitation. Hippocampus and prefrontal cortex (from the same animals), two brain regions with severe damages in the context of stress exposure (Bremner et al., 2000; Zlatković et al., 2014), were collected immediately. The total RNA in these tissues was extracted using an RNeasy mini kit according to manufacturer’s instructions (Qiagen, GmbH, Hilden, Germany). The first-strand of cDNA was generated by using a reverse transcription system (Promega, Madison, WI, United States). Real-time PCR was conducted with a reaction system containing 1 × Faststart SYBR Green Master Mix (Roche Molecular Biochemicals), 2 μL of diluted cDNA, 2 mM MgCl_2_, and 0.5 μM of primers. Primers for tumor necrosis factor-α (TNF-α), IL-1β, IL-6, IL-4, IL-10, Ym-1, and 18S rRNA are cited as follows (Gu et al., 2021): TNF-α, 5′-CTG​TGA​AGG​GAA​TGG​GTG​TT-3′ (F), 5′-GGTCAC TGTCCCAGCATCTT-3′ (R); IL-β, 5′-TGGAAAAGC GGTTTGTC TTC-3′ (F), 5′-TAC​CAG​TTG​GGG​AAC​TCT​GC-3′ (R); IL-6: 5′-AGA​GAT​ACA​AAG​AAA​TGA​TGG​A-3′ (F), 5′-AGC​TAT​GGT​ACT​CCA​CAA​GAC​CA-3′ (R); IL-4, 5′-CAG​CTA​GTT​GTC​ATC​CTG​CTC​TTC-3′ (F), 5′- GCC​GAT​GAT​CTC​TCT​CAA​GTG​A-3′ (R); IL-10, 5′-GGC​AGA​GAA​CCA​TGG​CCC​AGA​A-3′ (F), 5′-AAT​CGA​TGA​CAG​CGC​CTC​AGC​C-3′ (R); Ym-1, 5′-TCA​CTT​ACA​CAC​ATG​AGC​AAG​AC-3′ (F), 5′- CGG​TTC​TGA​GGA​GTA​GAG​ACC​A-3′ (R); 18S rRNA, 5′-GTA​ACC​CGT​TGA​ACC​CCA​TT-3′ (F), 5′-CCA​TCC​AAT​CGG​TAG​TAG​CG-3′ (R). PCR products were detected by monitoring the increase in intensity of fluorescence emitted by the double-stranded DNA-binding dye SYBR Green. An analysis of gene expression was performed by using the -ΔΔCt method. The values were normalized to the housekeeping gene 18S rRNA.

### Detection of Levels of Inflammatory Mediators

The levels of IL-1β, IL-6, TNF-α, IL-4, and IL-10 in the hippocampus and prefrontal cortex were determined according to manufacturer’s protocol available with their respective commercial kits (Proteintech, Wuhan, China). Concentrations of IL-1β, IL-6, TNF-α, IL-4, and IL-10 were expressed as picogram per Gram tissues (pg/g tissues).

### Detection of BDNF, Nitrite, Malondialdehyde, and Reduced Glutathione Contents

The levels of BDNF in the hippocampus and prefrontal cortex were measured using the BDNF DuoSet kit (R&D System; DY248) according to the manufacturer’s protocol. Briefly, a 96-well microplate were coated (overnight at room temperature) with 100 μl per well of a diluted capture antibody, followed by a careful wash on the second day. Next, the plates were blocked by using reagent diluent (300 μl, 1 h at room temperature). After that, 100 μL of sample or standards in reagent diluent was added into the prepared well and incubated at room temperature for 2 h. Then, the detection antibody (100 μl), the working dilution of streptavidin-HRP (100 μl), the substrate solution (100 μl), and the stop solution (50 μl) were added into each well in turn. Finally, the optical density of each well was determined using a microplate reader (Molecular Devices, Sunnyvale, CA, United States) set to 450 nm.

The levels of nitrite in the hippocampus and prefrontal cortex were measured according to the manufacturer’s protocol (Bi Yuntian Biological Technology Institution, Shanghai, China). Briefly, we first diluted the standard sample using a lysis buffer for the tissue containing 50 mM Tris-HCl (pH 7.4), 1 mM EDTA, 100 mM NaCl, 20 mM NaF, and 3 mM Na_3_VO_4_ with 1% NP-40, and then added the samples and reaction solutions into a 96-well microplate in the following order: 150 μl of samples (tissue supernatant in the lysis buffer), 20 μl of Griess Reagent, and 130 μl of de-ionized water. The nitrite concentration was determined by an M2 spectrophotometric microplate reader (548 nm, Molecular Devices, Sunnyvale, CA, United States) from a standard curve (0–100 μM/L) derived from NaNO_2_.

The contents of MDA and GSH were determined by the method used in one of our previous studies (Yang et al., 2017) by using the brain tissue homogenizations (10% w/v) in 0.1 M phosphate buffer (pH 7.4). The MDA and GSH contents were expressed as μM/g of wet tissue. The measurement of brain protein was according to the Lowry method (Lowry et al., 1951) and was expressed as μM/mg of protein concentration.

The tissue homogenizations from the mouse hippocampus and prefrontal cortex in all of the biochemical measurements were prepared using a Bioprep-24 Homogenizer (Aosheng, China).

### Statistical Analysis

Statistical analyses were performed using Graphpad Prism 8 (Graphpad Software, Inc., La Jolla, CA, United States). The pattern and distribution of our data were explored using the Kolmogorov-Smirnov test. Outliers were identified by the Grubbs’ outlier test and were excluded from analysis. Differences between the mean values of these data were evaluated using a two-way analysis of variance (ANOVA), and wherever necessary, Bonferroni’s post-hoc test was to assess isolated comparisons. When any two factors in an experiment did not interact in the ANOVA, we used a post hoc *t*-test, which was described previously by Wei et al. (2012), to make a further comparison between these factors, aiming to clarify whether the drug affect the CSDS-induced anxiety-like behaviors or the CSDS-induced production of anti-inflammatory cytokines. *p* values *<* 0.05 were considered statistically significant. Data are presented as mean ± standard error of mean (SEM).

## Results

### I3C Prevents CSDS-Induced Depression-Like Behaviors in Mice

We first evaluated the dose-dependent effect of I3C on CSDS-induced behavioral changes in the TST, SPT, FST, and SIT. The experimental procedure is outlined in Figure 1A. In the TST, the two-way ANOVA showed significant effects for stress exposure (F_1,72_ = 55.99, *p <* 0.001), drug treatment (F_3,72_ = 10.65, *p <* 0.001), and the stress × drug interaction (F_3,72_ = 5.07, *p <* 0.01) (Figure 1B). In the FST, the two-way ANOVA showed significant effects for stress exposure (F_1,72_ = 19.96, *p <* 0.001), drug treatment (F_3,72_ = 4.61, *p <* 0.01), and the stress × drug interaction (F_3,72_ = 4.97, *p <* 0.01) (Figure 1C). Post-hoc analysis revealed that I3C treatment during stress exposure at a dose of 10 mg/kg did not affect the CSDS-induced increases in the immobility time in the TST (Figure 1B) and FST (Figure 1C), but at a dose of 30 or 60 mg/kg, I3C treatment caused a significant suppression of the CSDS-induced increases in the immobility time in the TST (Figure 1B) and FST (Figure 1C). In the SPT, the two-way ANOVA showed significant effects for stress exposure (F_1,72_ = 52.74, *p <* 0.001), drug treatment (F_3,72_ = 5.83, *p <* 0.01), and the stress × drug interaction (F_3,72_ = 9.02, *p <* 0.001) (Figure 1D). Post-hoc analysis showed that I3C treatment at the dose of 30 and 60 mg/kg induced a marked suppression of the CSDS-induced decrease in sucrose intake in the SPT (Figure 1D).

In the SIT, a two-way ANOVA for the time spent in the interaction zone when the target was absent showed no significant effects for stress exposure (F_1,72_ = 0.03, *p* = 0.86), different doses of drug treatment (F_3,72_ = 0.13, *p* = 0.94), and the stress × drug interaction (F_3,72_ = 0.55, *p* = 0.65) (Figure 1E), while when the target was present the two-way ANOVA showed significant effects for the time spent in the interaction zone for stress exposure (F_1,72_ = 16.50, *p <* 0.001), drug treatment (F_3,72_ = 2.82, *p <* 0.05), and the stress × drug interaction (F_3,72_ = 3.52, *p <* 0.05) (Figure 1F). Post-hoc analysis revealed that I3C treatment at a dose of 10 mg/kg did not affect the CSDS-induced reduction in the time spent in the interaction zone in the SIT (Figure 1F). At a dose of 30 mg/kg, I3C treatment prevented the CSDS-induced reduction in the time spent in the interaction zone in the SIT (Figure 1F). I3C treatment at the dose of 60 mg/kg produced similar effects against CSDS-induced reductions of the time spent in the interaction zone in the SIT (Figure 1F). Further analysis showed that the injection of I3C at the dose of 10, 30, and 60 mg/kg did not induce significant changes in behavioral changes in TST (Figure 1B), FST (Figure 1C), SPT (Figure 1D), and SIT (Figures 1E,F) in stress-naïve mice. These results demonstrated that I3C treatment can prevent the development of depression-like behaviors in chronically-stressed mice in a dose-dependent manner. As the effect of IC3 treatment at the dose of 60 mg/kg appeared to be better than that observed at the dose of 30 mg/kg, the 60 mg/kg was selected for further analysis.

### I3C Does not Affect CSDS-Induced Anxiety-Like Behaviors in Mice

We next evaluated the effect of I3C on CSDS-induced anxiety-like behaviors in mice (Figure 2A). In the EPM test, the two-way ANOVA for the time spent in the open arms showed a significant effect for stress exposure (F_1,72_ = 140.10, *p <* 0.001), but not for drug treatment (F_3,72_ = 0.15, *p =* 0.93) and the stress × drug interaction (F_3,72_ = 0.37, *p =* 0.77) (Figure 2B). Post-hoc analysis revealed that I3C treatment at the dose of 10, 30, and 60 mg/kg did not prevent the CSDS-induced decrease in the time spent in open arms in the EPM test (Figure 2B). In the LDT test, the two-way ANOVA for the time spent in lit side showed a significant effect for stress exposure (F_1,72_ = 163.80, *p <* 0.001), but not for drug treatment (F_3,72_ = 0.20, *p =* 0.89) and the stress × drug interaction (F_3,72_ = 0.49, *p =* 0.69) (Figure 2C). Post-hoc analysis revealed that I3C treatment at the dose of 10, 30, and 60 mg/kg did not prevent the CSDS-induced decrease in the time spent in lit side in the LDT (Figure 2C). In the OFT, the two-way ANOVA for the time spent in the central region of the open field showed a significant effect for stress exposure (F_1,72_ = 99.39, *p <* 0.001), but not for drug treatment (F_3,72_ = 0.26, *p =* 0.85) and the stress × drug interaction (F_3,72_ = 0.13, *p =* 0.94) (Figure 2D). Post-hoc analysis revealed that I3C treatment at the dose of 10, 30, and 60 mg/kg did not prevent the CSDS-induced decrease in the time spent in the central region of the open field in the OFT (Figure 2D). Further analysis showed that both CSDS and/or I3C treatment (10, 30, and 60 mg/kg) did not affect the total distance of mice in the OFT, with no significant effects for stress exposure (F_1,72_ = 0.04, *p =* 0.84), drug treatment (F_3,72_ = 0.19, *p =* 0.91), and the stress × drug interaction (F_3,72_ = 0.47, *p =* 0.70) in the two-way ANOVA (Figure 2E).

As there was no significant stress × drug interaction, and the main effect of stress exposure was significant, while the main effect of drug treatment was not significant, we conducted a further analysis using a method that is based on the *t*-test described in a previous study by Wei et al. (2012) to determine if there was any difference between the tested values of the vehicle and CSDS/I3C group in the EPM, LDT, and OFT. This information was essential as one the major aims of the present study is to determine whether repeated I3C supplementation could prevent CSDS-induced anxiety-like behaviors in mice. The post-hoc *t*-test showed that there was a significant difference between the tested values of the vehicle and CSDS/I3C group in the EPM (Figure 2B), LDT (Figure 2C), and OFT (Figure 2D). These results demonstrate that repeated I3C treatment at any selected doses (10, 30, and 60 mg/kg) used in this study did not prevent CSDS-induced depression-like behaviors.

### I3C Prevents CSDS-Induced Increases in Levels Pro-Inflammatory Cytokine mRNA and Protein in the Hippocampal and Prefrontal Cortex

**FIGURE 3:**
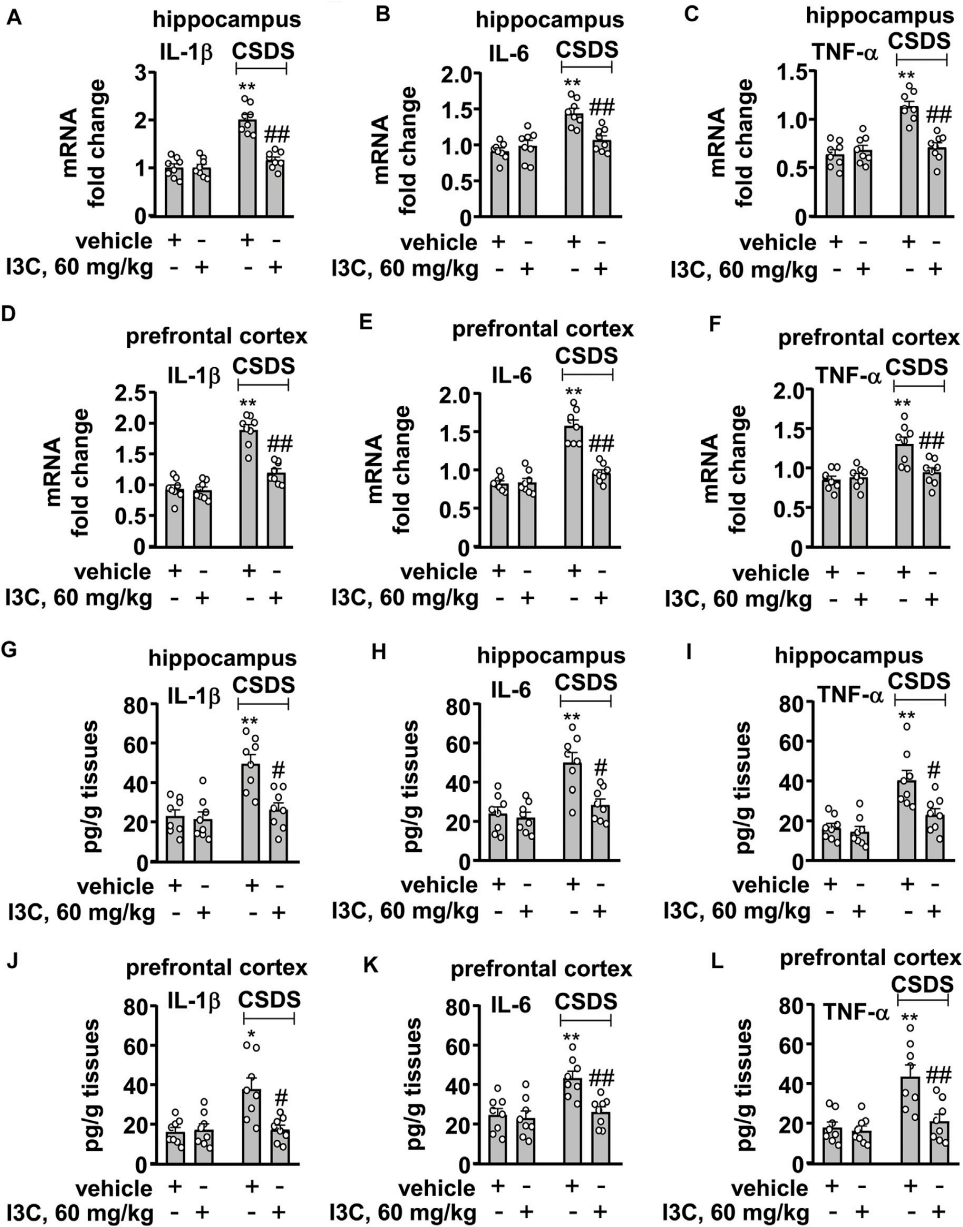
Effect of I3C treatment on CSDS-induced increases in pro-inflammatory cytokines in the hippocampus and prefrontal cortex in mice. **(A‒F)** Quantitative analysis showed that I3C treatment (60 mg/kg) prevented the CSDS-induced increases in the expression levels of IL-1β [**(A)**, hippocampus; **(D)**, cortex], IL-6 [**(B)**, hippocampus; **(E)**, cortex], and TNF-α [**(C)**, hippocampus; **(F)**, cortex] mRNA in the hippocampus and prefrontal cortex (*n* = 8, **p < 0.01 vs. vehicle; ##*p* < 0.01 vs. vehicle + CSDS). (**G‒L**) Quantitative analysis showed that I3C treatment (60 mg/kg) prevented the CSDS-induced increases in levels IL-1β [**(G)**, hippocampus; **(J)**, cortex], IL-6 [**(H)**, hippocampus; **(K)**, cortex)], and TNF-α [**(I)**, hippocampus; **(L)**, cortex)] protein in the hippocampus and prefrontal cortex (*n* = 8, *p < 0.05 or **p < 0.01 vs. vehicle; #*p* < 0.05 or ##*p* < 0.01 vs. vehicle + CSDS). Data are shown as mean ± SEM.

Next, we evaluated the influence of I3C treatment on the levels of pro-inflammatory cytokine mRNA and protein in the hippocampus and prefrontal cortex in mice treated with or without CSDS. A two-way ANOVA for the levels of IL-1β, IL-6, and TNF-α mRNA in the hippocampus showed significant effects for stress exposure (IL-1β: F_1,28_ = 55.81, *p* < 0.001, IL-6: F_1,28_ = 25.47, *p* < 0.001, TNF-α: F_1,28_ = 29.35, *p* < 0.001), drug treatment (IL-1β: F_1,28_ = 29.46, *p* < 0.001; IL-6: F_1,28_ = 6.10, *p* < 0.05; TNF-α: F_1,28_ = 15.37, *p* < 0.001), and the stress × drug interaction (IL-1β: F_1,28_ = 29.96, *p* < 0.001; IL-6: F_1,28_ = 13.60, *p* < 0.001; TNF-α: F_1,28_ = 23.51, *p* < 0.001) (Figures 3A–C). Post-hoc analysis revealed that I3C treatment at the dose of 60 mg/kg prevented the CSDS-induced increases in the expression levels of IL-1β (Figure 3A), IL-6 (Figure 3B), and TNF-α (Figure 3C) mRNA in the hippocampus.

For the levels of IL-1β, IL-6, and TNF-α mRNA in the prefrontal cortex, the ANOVA showed significant effects for stress exposure (IL-1β: F_1,28_ = 89.99, *p* < 0.001, IL-6: F_1,28_ = 69.40, *p* < 0.001, TNF-α: F_1,28_ = 18.19, *p* < 0.001), drug treatment (IL-1β: F_1,28_ = 29.57, *p* < 0.001; IL-6: F_1,28_ = 33.28, *p* < 0.05; TNF-α: F_1,28_ = 7.37, *p* < 0.05), and the stress × drug interaction (IL-1β: F_1,28_ = 22.27, *p* < 0.001; IL-6: F_1,28_ = 35.80, *p* < 0.001; TNF-α: F_1,28_ = 10.53, *p* < 0.01) (Figures 3D–F). Post-hoc analysis showed that I3C treatment at the dose of 60 mg/kg prevented the CSDS-induced increases in the expression levels of IL-1β (Figure 3D), IL-6 (Figure 3E), and TNF-α (Figure 3F) mRNA in the prefrontal cortex.

The two-way ANOVA for the levels of IL-1β, IL-6, and TNF-α protein in the hippocampus showed significant effects for stress exposure (IL-1β: F_1,28_ = 17.45, *p* < 0.001, IL-6: F_1,28_ = 20.17, *p* < 0.001, TNF-α: F_1,28_ = 23.66, *p* < 0.001), drug treatment (IL-1β: F_1,28_ = 11.17, *p* < 0.01; IL-6: F_1,28_ = 10.64, *p* < 0.01; TNF-α: F_1,28_ = 8.32, *p* < 0.01), and the stress × drug interaction (IL-1β: F_1,28_ = 8.54, *p* < 0.01; IL-6: F_1,28_ = 7.34, *p* < 0.05; TNF-α: F_1,28_ = 5.38, *p* < 0.05) (Figures 3D–F). Post-hoc analysis revealed that I3C treatment at the dose of 60 mg/kg prevented the CSDS-induced increases in the expression levels of IL-1β (Figure 3D), IL-6 (Figure 3E), and TNF-α (Figure 3F) protein in the hippocampus.

For the levels of IL-1β, IL-6, and TNF-α protein in the prefrontal cortex, the ANOVA showed significant effects for stress exposure (IL-1β: F_1,28_ = 9.76, *p* < 0.01, IL-6: F_1,28_ = 11.69, *p* < 0.01, TNF-α: F_1,28_ = 15.56, *p* < 0.001), drug treatment (IL-1β: F_1,28_ = 7.70, *p* < 0.01; IL-6: F_1,28_ = 8.75, *p* < 0.01; TNF-α: F_1,28_ = 9.89, *p* < 0.01), and the stress × drug interaction (IL-1β: F_1,28_ = 9.55, *p* < 0.01; IL-6: F_1,28_ = 6.14, *p* < 0.05; TNF-α: F_1,28_ = 7.50, *p* < 0.05) (Figures 3G–I). Post-hoc analysis showed that I3C treatment at the dose of 60 mg/kg prevented the CSDS-induced increases in the expression levels of IL-1β (Figure 3G), IL-6 (Figure 3H), and TNF-α (Figure 3I) protein in the prefrontal cortex.

### I3C Does not Affect CSDS-Induced Decreases in Anti-Inflammatory Mediators in the Hippocampal and Prefrontal Cortex

**FIGURE 4:**
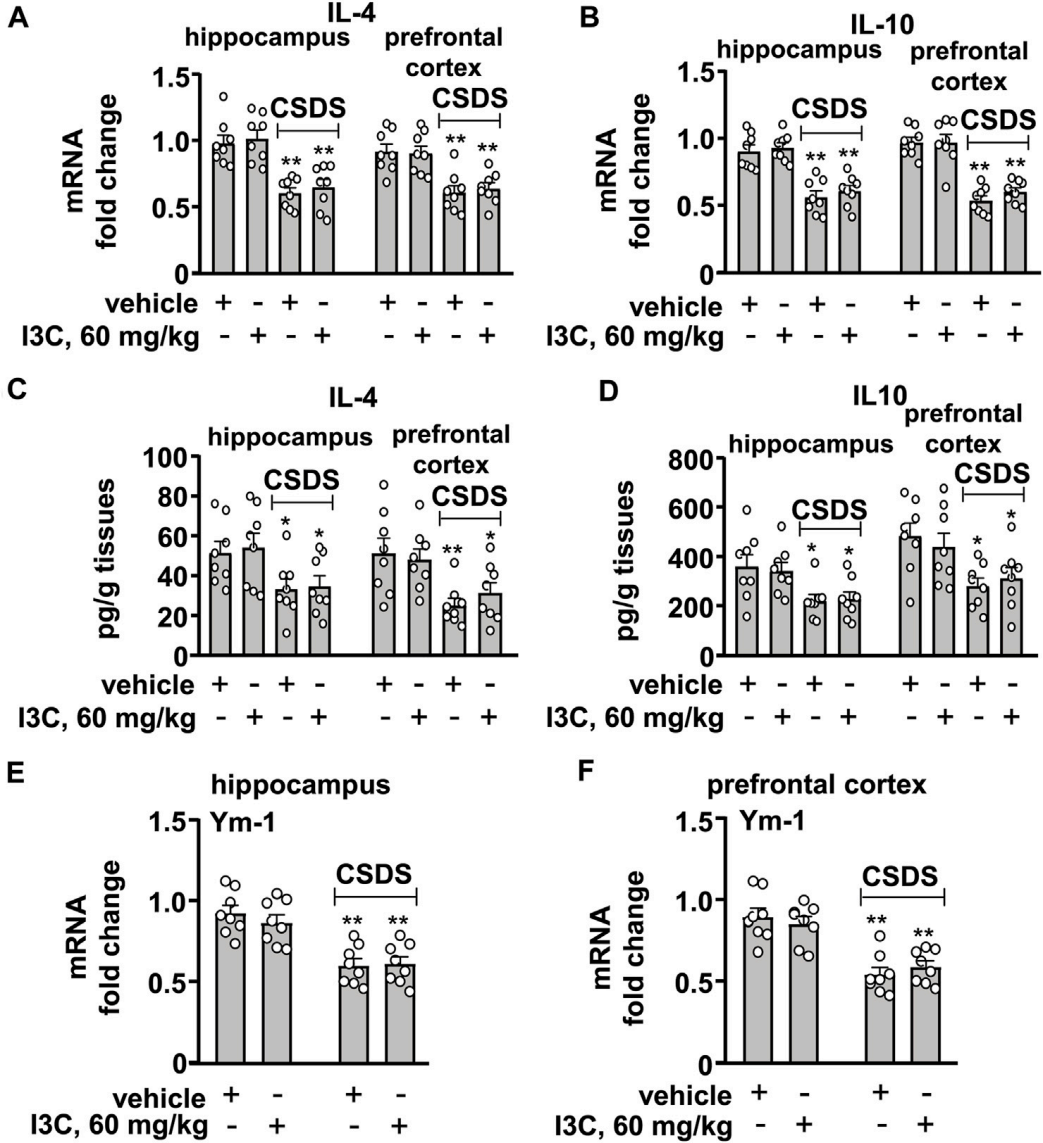
Effect of I3C treatment on CSDS-induced decreases in anti-inflammatory cytokines in the hippocampus and prefrontal cortex in mice. **(A,B)** Quantitative analysis showed that I3C treatment (60 mg/kg) did not prevent the CSDS-induced decreases in the expression levels of IL-4 **(A)** and IL-10 **(B)** mRNA in the hippocampus and prefrontal cortex (*n* = 8, **p < 0.01 vs. vehicle). **(C,D)** Quantitative analysis showed that I3C treatment (60 mg/kg) during stress exposure did not prevent the CSDS-induced decreases in levels of IL-4 **(A)** and IL-10 **(B)** protein in the hippocampus and prefrontal cortex (*n* = 8, *p < 0.05 or **p < 0.01 vs. vehicle). **(E,F)** Quantitative analysis showed that I3C treatment (60 mg/kg) did not prevent the CSDS-induced decreases in the expression levels of Ym-1 mRNA in the hippocampus **(E)** and prefrontal cortex **(F)** (*n* = 8, **p < 0.01 vs. vehicle). Data are shown as mean ± SEM.

We also evaluated the effect of I3C treatment on anti-inflammatory cytokines in the hippocampus and prefrontal cortex in chronically stressed mice. For the expression levels of IL-4 mRNA in the hippocampus and prefrontal cortex, a two-way ANOVA showed a significant effect for stress exposure (hippocampus: F_1,28_ = 44.31, *p* < 0.001, cortex: F_1,28_ = 31.19, *p* < 0.001), but not for drug treatment (hippocampus: F_1,28_ = 0.51, *p* = 0.48; cortex: F_1,28_ = 0.03, *p* = 0.85) and the stress × drug interaction (hippocampus: F_1,28_ = 0.007, *p* = 0.93; cortex: F_1,28_ = 0.18, *p* = 0.68) (Figure 4A), and for the expression levels of IL-10 mRNA the ANOVA showed a significant effect for stress exposure (hippocampus: F_1,28_ = 61.79, *p* < 0.001, cortex: F_1,28_ = 92.59, *p* < 0.001), but not for drug treatment (hippocampus: F_1,28_ = 0.70, *p* = 0.41; cortex: F_1,28_ = 0.60, *p* = 0.45) and the stress × drug interaction (hippocampus: F_1,28_ = 0.07, *p* = 0.80; cortex: F_1,28_ = 0.65, *p* = 0.43) (Figure 4B). Post-hoc analysis revealed that I3C treatment at the dose of 60 mg/kg did not prevent the CSDS-induced decreases in the expression levels of IL-4 (Figure 4A) and IL-10 (Figure 4B) mRNA in the hippocampus and prefrontal cortex.

For the levels of IL-4 protein in the hippocampus and prefrontal cortex, the two-way ANOVA showed a significant effect for stress exposure (hippocampus: F_1,28_ = 10.54, *p* < 0.01, cortex: F_1,28_ = 15.00 *p* < 0.001), but not for drug treatment (hippocampus: F_1,28_ = 0.13, *p* = 0.72; cortex: F_1,28_ = 0.06, *p* = 0.80) and the stress × drug interaction (hippocampus: F_1,28_ = 0.009, *p* = 0.92; cortex: F_1,28_ = 0.75, *p* = 0.40) (Figure 4C), and for the levels of IL-10 protein the ANOVA showed a significant effect for stress exposure (hippocampus: F_1,28_ = 13.17, *p* < 0.01, cortex: F_1,28_ = 12.85, *p* < 0.01), but not for drug treatment (hippocampus: F_1,28_ = 0.02, *p* = 0.90; cortex: F_1,28_ = 0.02, *p* = 0.88) and the stress × drug interaction (hippocampus: F_1,28_ = 0.13, *p* = 0.72; cortex: F_1,28_ = 0.65, *p* = 0.42) (Figure 4D). Post-hoc analysis revealed that I3C treatment at the dose of 60 mg/kg did not prevent the CSDS-induced decreases in levels of IL-4 (Figure 4C) and IL-10 (Figure 4D) protein in the hippocampus and prefrontal cortex.

For the levels of Ym-1 mRNA in the hippocampus and prefrontal cortex, the two-way ANOVA showed a significant effect for stress exposure (hippocampus: F_1,28_ = 40.36, *p* < 0.001; cortex: F_1,28_ = 48.93, *p* < 0.001), but not for drug treatment (hippocampus: F_1,28_ = 0.28, *p* = 0.60; cortex: F_1,28_ = 0.004, *p* = 0.95) and the stress × drug interaction (hippocampus: F_1,28_ = 0.56, *p* = 0.46; cortex: F_1,28_ = 1.00, *p* = 0.44) (Figures 4E,F). Post-hoc analysis revealed that I3C treatment at the dose of 60 mg/kg also did not prevent the CSDS-induced decreases in the expression levels of Ym-1 mRNA in the hippocampus (Figure 4E) and prefrontal cortex (Figure 4F).

As the statistical analysis showed no significant stress × drug interaction, and the main effect of stress exposure was significant, while the main effect of drug treatment was not, we used the *t*-test to determine if there was any difference between the tested values of the vehicle and CSDS/I3C group in these experiments. The post-hoc *t*-test showed that there was a significant difference between the tested values of the vehicle and CSDS/I3C group for the expression levels of IL-4 mRNA (Figure 4A), IL-10 mRNA (Figure 4B), IL-4 protein (Figure 4C), IL-10 protein (Figure 4D), Ym-1 mRNA (Figures 4E,F) in the hippocampus and prefrontal cortex.

### I3C Prevents CSDS-Induced Changes in Markers That Indicate Oxido-Nitrosative Stress in the Hippocampus and Prefrontal Cortex

**FIGURE 5:**
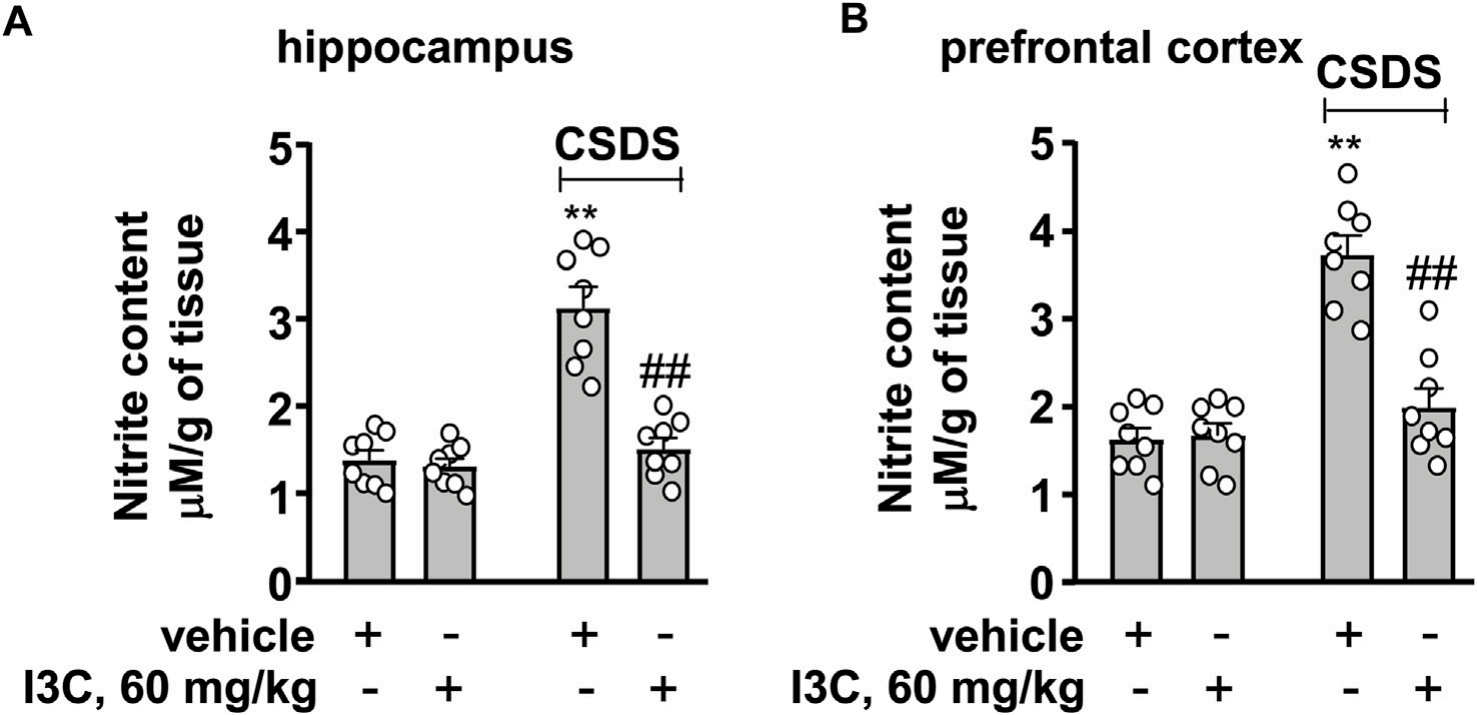
Effect of I3C on CSDS-induced increases in nitrite levels in the hippocampus and prefrontal cortex in mice. **(A,B)** Quantitative analysis showed that I3C treatment (60 mg/kg) prevented the CSDS-induced increases in levels of nitrite in the hippocampus **(A)** and prefrontal cortex **(B)** (*n* = 8, **p < 0.01 vs. vehicle; ##*p* < 0.01 vs. vehicle + CSDS). Data are shown as mean ± SEM.

We then evaluated the effect of I3C treatment on markers that indicate oxido-nitrosative stress in the hippocampus and prefrontal cortex in chronically stressed mice. A two-way ANOVA for levels of nitrite in the hippocampus and prefrontal cortex showed significant effects for stress exposure (hippocampus: F_1,28_ = 44.70, *p* < 0.001, cortex: F_1,28_ = 48.85, *p* < 0.001), drug treatment (hippocampus: F_1,28_ = 33.41, *p* < 0.001, cortex: F_1,28_ = 23.60, *p* < 0.001), and the stress × drug interaction (hippocampus: F_1,28_ = 28.08, *p* < 0.001, cortex: F_1,28_ = 26.44, *p* < 0.001) (Figures 5A,B). Post-hoc analysis revealed that I3C treatment at the dose of 60 mg/kg prevented the CSDS-induced increases in nitrite levels in the hippocampus and prefrontal cortex (Figures 5A,B).

**FIGURE 6:**
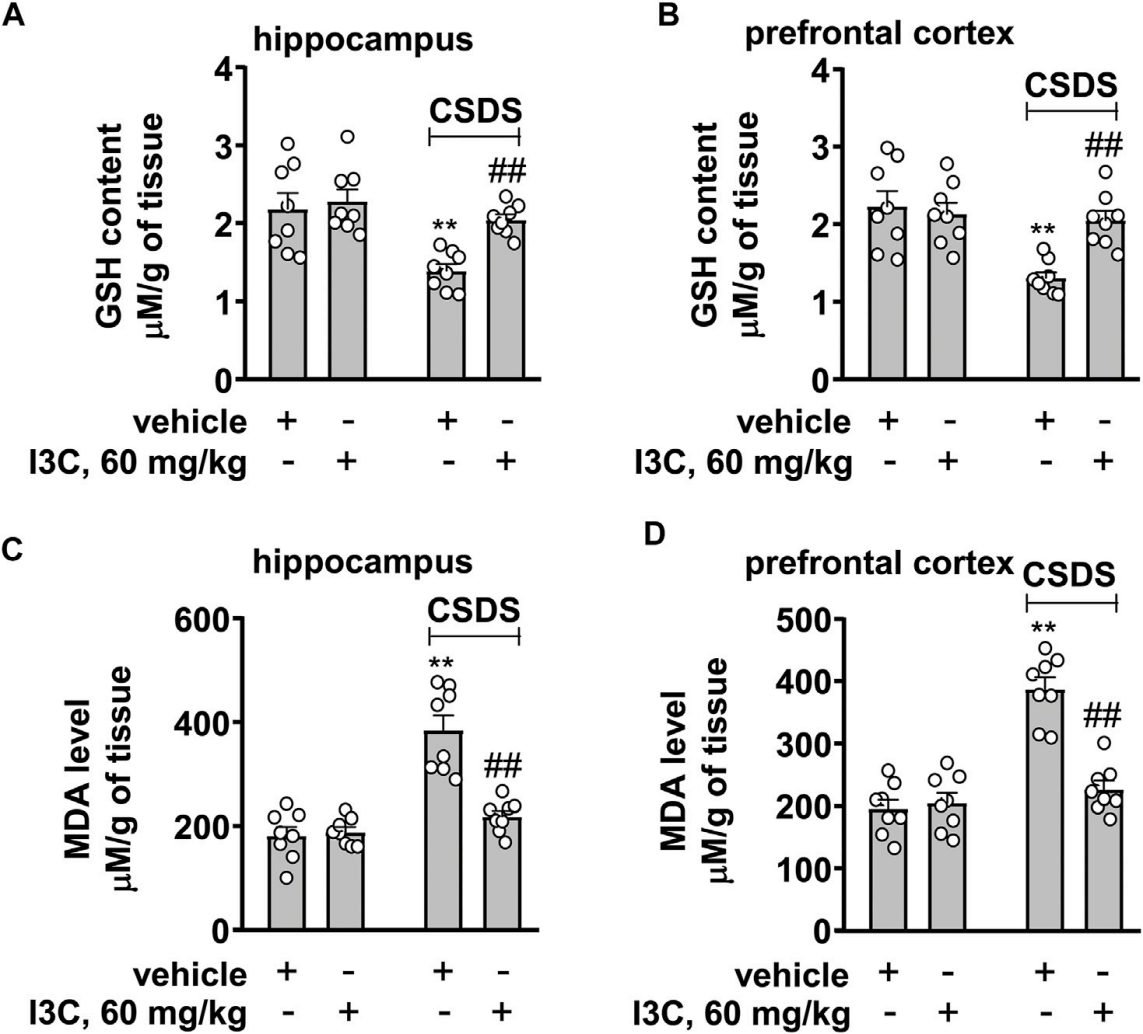
Effect of I3C on CSDS-induced changes in contents of GSH and MDA in the hippocampus and prefrontal cortex in mice. **(A,B)** Quantitative analysis showed that I3C treatment (60 mg/kg) prevented the CSDS-induced decreases in contents of GSH in the hippocampus and prefrontal cortex (*n* = 8, **p < 0.01 vs. vehicle; ##*p* < 0.01 vs. vehicle + CSDS). **(C,D)** Quantitative analysis showed that I3C treatment (60 mg/kg) prevented the CSDS-induced increases in contents of MDA in the hippocampus and prefrontal cortex (*n* = 8, **p < 0.01 vs. vehicle; ##*p* < 0.01 vs. vehicle + CSDS). Data are shown as mean ± SEM.

We also evaluated the effect of I3C treatment on contents of GSH and MDA in the hippocampus and prefrontal cortex in chronically stressed mice. A two-way ANOVA for GSH in the hippocampus and prefrontal cortex showed significant effects for stress exposure (hippocampus: F_1,28_ = 14.42, *p* < 0.001, cortex: F_1,28_ = 12.73, *p* < 0.01), drug treatment (hippocampus: F_1,28_ = 7.58, *p* < 0.05, cortex: F_1,28_ = 5.24, *p* < 0.05), and the stress × drug interaction (hippocampus: F_1,28_ = 4.23, *p* < 0.05, cortex: F_1,28_ = 8.90, *p* < 0.01) (Figures 6A,B). Post-hoc analysis revealed that I3C treatment at the dose of 60 mg/kg prevented the CSDS-induced decreases in GSH contents in the hippocampus (Figure 6A) and prefrontal cortex (Figure 6B).

For MDA contents in the hippocampus and prefrontal cortex, the ANOVA showed significant effects for stress exposure (hippocampus: F_1,28_ = 42.44, *p* < 0.001, cortex: F_1,28_ = 45.57, *p* < 0.01), drug treatment (hippocampus: F_1,28_ = 19.85, *p* < 0.001, cortex: F_1,28_ = 22.83, *p* < 0.001), and the stress × drug interaction (hippocampus: F_1,28_ = 23.45, *p* < 0.001, cortex: F_1,28_ = 29.15, *p* < 0.001) (Figures 6C,D). Post-hoc analysis revealed that I3C treatment at the dose of 60 mg/kg prevented the CSDS-induced increases in MDA contents in the hippocampus (Figure 6C) and prefrontal cortex (Figure 6D).

### I3C Prevents CSDS-Induced Decreases in BDNF in the Hippocampal and Prefrontal Cortex

**FIGURE 7:**
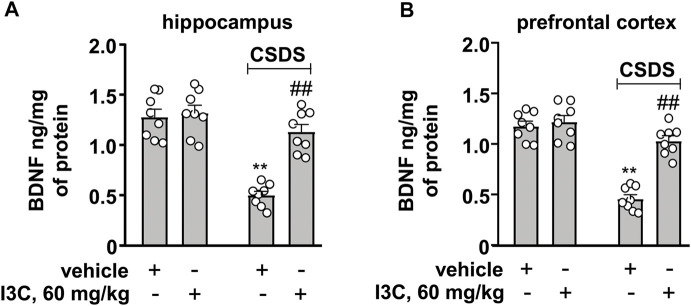
Effect of I3C on CSDS-induced increases in levels of BDNF in the hippocampus and prefrontal cortex in mice. **(A, B)** Quantitative analysis showed that I3C treatment (60 mg/kg) prevented the CSDS-induced decreases in levels of BDNF protein in the hippocampus **(A)** and prefrontal cortex **(B)** (*n* = 8, **p < 0.01 vs. vehicle; ##*p* < 0.01 vs. vehicle + CSDS). Data are shown as mean ± SEM.

Finally, we evaluated the effect of I3C on BDNF contents in the hippocampus and prefrontal cortex in chronically stressed mice. A two-way ANOVA for BDNF contents in the hippocampus and prefrontal cortex showed significant effects for stress exposure (hippocampus: F_1,28_ = 49.23, *p* < 0.001, cortex: F_1,28_ = 78.78, *p* < 0.01), drug treatment (hippocampus: F_1,28_ = 23.89, *p* < 0.001, cortex: F_1,28_ = 36.32, *p* < 0.001), and stress × drug interaction (hippocampus: F_1,28_ = 18.57, *p* < 0.001, cortex: F_1,28_ = 26.95, *p* < 0.001) (Figures 7A,B). Post-hoc analysis revealed that I3C treatment at the dose of 60 mg/kg prevented the CSDS-induced decreases in BDNF contents in the hippocampus (Figure 7A) and prefrontal cortex (Figure 7B).

## Discussion

One of the major findings in the present study was that I3C, a plant-derived compound, administered simultaneously with stress stimulation, produced inhibitory effects on CSDS-induced depression-like behaviors in the TST, FST, SPT, and SIT in mice in a dose-dependent manner. This demonstrated that I3C could be applied to prevent the development of depression-like behaviors in animals. As CSDS is also known to induce anxiety-like behaviors, we supposed that repeated I3C treatment may prevent chronic stress-induced anxiety-like behaviors. However, we found that I3C, administered repeatedly at the dose of 10, 30, or 60 mg/kg, did not prevent CSDS-induced decreases in the time spent 1) in open arms in the EPM test, 2) in lit side in the LDT, and 3) in the central area of the open field in the OFT. These findings demonstrated that I3C supplementation may be not effective in preventing the development of anxiety-like behaviors in chronically stressed mice. Thus, it is reasonable to conclude that supplementation of I3C or I3C-containing plants could be a potential strategy for the prevention of depression, but not anxiety, under stresses conditions. However, the exact reasons for this differential effect of I3C remain unclear. It should be clarified by more studies.

In clinic, besides the traditional antidepressants, little drugs could be available for the treatment of depression, which could be due to the deficiency of the understanding about the pathological mechanisms of depression. In recent years, the over-accumulation of pro-inflammatory cytokines in the blood and brain tissues has been widely-reported to be associated with the pathogenesis of depression. For example, the over-activation of microglia has been repeatedly reported to mediate the progression of depression in rodent animals (Zhao et al., 2016; Duan et al., 2020; Jing Li et al., 2020; Wu et al., 2020; Kumar et al., 2021), and suppression of neuroinflammation by minocycline treatment could can ameliorate depressive symptoms (Wang et al., 2020; Bassett et al., 2021). Individuals suffering from depression have increased levels of pro-inflammatory cytokines in the blood (Petralia et al., 2020; Primo de Carvalho Alves and Sica da Rocha, 2020). Administration of clinically-available antidepressants can down-regulate the levels of pro-inflammatory cytokines in the blood in depressed patients and in brain tissues in animals (Du et al., 2016; Lu et al., 2019; Wang et al., 2019). Thus, inhibition of the neuroinflammation is a promising strategy for the prevention and/or treatment of depression. Our results showed that the repeated I3C treatment during the 10-days of defeat stress exposure suppressed the CSDS-induced increases in the mRNA and protein levels of IL-1β, IL-6, and TNF-α in the hippocampus and prefrontal cortex, suggesting that I3C supplementary may exhibit strong anti-neuroinflammatory activities in animal models of depression.

It is known that the development of neuroinflammatory responses at stress conditions is usually accompanied with significant reductions in anti-inflammatory mediators (Zhao et al., 2016; Duan et al., 2020; Jing Li et al., 2020; Wu et al., 2020; Kumar et al., 2021). Thus, enhancing the production of anti-inflammatory mediators in the brain may mediate the antidepressant effect of I3C. However, our results showed that repeated I3C treatment did not prevent the CSDS-induced decreases in the mRNA and/or protein levels of anti-inflammatory mediators in the brain, suggesting that the antidepressant-like effect of I3C may be not associated with the increases in anti-inflammatory mediators. Generally, these results demonstrated that I3C supplementation may selectively suppress the development of depression-like behaviors in CSD mice via preventing the over-production of pro-inflammatory cytokines in the brain. However, it is worth pointing out that the herein-observed effect of I3C on pro-inflammatory cytokines may not support a causal relationship between the anti-neuroinflammatory and the antidepressant-like effect of I3C, as besides the neuroinflammatory theory there are many other hypotheses that could be used to explain the pathogenesis of depression (Spellman and Liston, 2020; Tubbs et al., 2020). Our results only revealed a possible contribution of anti-neuroinflammation to the antidepressant-like effect of I3C. More studies should be done to clarify the exact mechanisms underlying the antidepressant-like effect of I3C.

In the pathogenesis of depression, the increased pro-inflammatory cytokines in the brain are known to impair neuronal functions by down-regulating the expression of BDNF (Cheng et al., 2019). Inhibition of the progression of neuroinflammation could be accompanied with an enhancement of BDNF expression (Li et al., 2021). Our results showed that I3C supplementation could prevent the CSDS-induced decreases in BDNF contents in the hippocampus and prefrontal cortex, suggesting that the increased BDNF upon repeated I3C treatment could be due to pro-inflammatory cytokine decrease. The increased pro-inflammatory cytokines can also induce a cascade involving oxido-nitrosative stress, during which the increased nitrite together with the reactive oxygen species mediates the progression of depression likely through down-regulation of BDNF expression (Cheng et al., 2019; Lima Giacobbo et al., 2019; Kangwei Li et al., 2020). Thus, searching drugs that can suppress oxido-nitrosative stress may be an attractive strategy for the treatment of depression. Our results showed that repeated I3C treatment prevented the CSDS-induced increases in MDA and nitrite levels and decreases in GSH contents in the hippocampus and prefrontal cortex, suggesting that besides overcoming the overactivated neuroinflammatory response, protecting against oxido-nitrosative stress could be another potential mechanism for the antidepressant-like effect of I3C.

As the oxido-nitrosative stress and the impairment of the BDNF signal in the brain are also known to mediate the pathogenesis of anxiety (Li et al., 2017; Olugbemide et al., 2021), our findings about the regulatory effect of I3C on oxido-nitrosative stress and the BDNF signal should reveal a possible role of I3C in amelioration of anxiety; however, the fact is that the I3C supplementation did not prevent the CSDS-induced anxiety-like behaviors in mice. Thus, it is reasonable to conclude that under stress conditions, the increased pro-inflammatory cytokines and oxidative-nitrosative stress and the impairment of the BDNF signal, at least in the hippocampus and prefrontal cortex, may not contribute to the progression of anxiety-like behaviors in chronically stressed animals. Although the pathogenesis of anxiety involves a variety of biological processes and brain regions, the failure of I3C to prevent the occurrence of anxiety-like behaviors in CSDS mice may provide a great chance to re-visit the pathogenesis of anxiety.

## Conclusion

Our results showed that repeated I3C treatment selectively prevented the CSDS-induced depression- but not anxiety-like behaviors in mice, with an attenuation of neuroinflammation and oxido-nitrosative stress as well as an increase in BDNF levels in the brain. These findings may help to develop drugs with specific effects on depression. However, it is necessary to point out that we still do not know why the repeated I3C treatment specifically prevents depression-like behaviors as well as the production of pro-inflammatory cytokines in the brain. In future studies, we should clarify the roles of the targets that mediate the pharmacological effects of I3C, such as the neural precursor cell expressed, developmentally down-regulated 4, E3 ubiquitin protein ligase (Nedd4-1) and aryl hydrocarbon receptor (AHR) (Aronchik et al., 2014; Mohammadi et al., 2018), in the herein-observed effect of I3C.

## Data Availability

The raw data supporting the conclusions of this article will be made available by the authors, without undue reservation.
